# Prognostic value of neutrophil extracellular trap signature in clear cell renal cell carcinoma

**DOI:** 10.3389/fonc.2023.1205713

**Published:** 2023-07-13

**Authors:** Rong Li, Xuewen Jiang, Pin Wang, Xiaoyan Liu

**Affiliations:** ^1^ Phase I Clinical Trial Center, Qilu Hospital of Shandong University, Jinan, China; ^2^ Department of Urology, Qilu Hospital of Shandong University, Jinan, China; ^3^ National Health Commission (NHC) Key Laboratory of Otorhinolaryngology, Department of Otorhinolaryngology, Qilu Hospital of Shandong University, Shandong University, Jinan, Shandong, China

**Keywords:** clear cell renal cell carcinoma, neutrophil extracellular traps, prognosis signature, validation, multiomics data, immune microenvironment, therapy sensitivity

## Abstract

**Introduction:**

Clear cell renal cell carcinoma (ccRCC) is the most prevalent type of urological carcinoma. Although targeted therapy and immunotherapy are usually employed, they often result in primary and acquired resistance. There is currently a lack of dependable biomarkers that can accurately anticipate the prognosis of ccRCC. Recent research has indicated the critical role of neutrophil extracellular traps (NETs) in the development, metastasis, and immune evasion of cancer. The aim of this study was to explore the value of NETs in the development and prognosis of ccRCC.

**Methods:**

Clinical features and genetic expression information of ccRCC patients were acquired from The Cancer Genome Atlas (TCGA), International Cancer Genome Consortium (ICGC) and E-MTAB-1980 database. NETs-related gene set were obtained from previous studies. A NETs-related gene signature was constructed based on TCGA data and validated using ICGC and E-MTAB-1980 databases. Furthermore, the immune microenvironment and responsiveness to anticancer medications in ccRCC patients with varying levels of NETs risks were investigated.

**Results:**

A total of 31 NET-related genes were differently expressed between normal kidney and ccRCC tissues. 17 out of 31 were significantly associated with overall survival. After LASSO Cox regression analysis, nine NETs-related genes were enrolled to construct the NETs prognosis signature, and all the ccRCC patients from TCGA were divided into low and high risk group. This signature demonstrated excellent performance in predicting the overall survival of TCGA patients as well as the validation ICGC and E-MTAB-1980 patients. Additionally, the NETs signature was significantly correlated with immune infiltration and drug sensitivity.

**Conclusions:**

The NETs signature established by the current study has prognostic significance in ccRCC, and may serve as a useful biomarker for patient stratification and treatment decisions. Further validation and clinical studies are required to fully translate these findings into clinical practice.

## 1 Introduction

Renal cell carcinoma (RCC) is the most prevalent type of kidney cancer, accounting for almost 2% of all cancer diagnoses and cancer deaths (1). Worldwide, the incidence of RCC is increasing annually and varies per region, with generally higher incidence rates in developed countries than in developing countries (2). RCC consists of a heterogeneous group of cancer with distinct genetic and molecular variability. Approximately 70% to 75% of all cases of RCC belong to the sub-type known as clear cell RCC (ccRCC). For patients with localized RCC, partial or radical nephrectomy is the recommended curative treatment. On the other hand, systemic therapy is the remaining treatment option for patients suffering from metastatic RCC. Unlike other tumor types, RCC does not respond to conventional chemotherapeutic agents or radiotherapy (3). Despite the breakthrough advances in targeted drugs and immunotherapy in recent years, drug resistance and restricted progression-free survival continue to persist. This is especially true for patients with metastatic ccRCC (4). Although certain pathological biomarkers and clinical indicators have been employed to anticipate the prognosis of ccRCC and treatment outcome, their predictive capability is insufficient due to significant inter-individual variation (5, 6). As a result, it is urgent to identify novel therapeutic targets to improve the prognosis of ccRCC.

Neutrophil extracellular traps (NETs) are structures made up of DNA, histones, proteases and proteins which are released by activated neutrophils (7). NETosis is the process of typical NETs formation and is deemed a distinct kind of controlled cellular demise, separate from apoptosis, autophagy or necroptosis (8, 9). In recent years, the role of NETs in cancer has become a topic of interest, as they have been implicated in the regulation of tumor progression, metastasis and recurrence (10, 11). Moreover, the involvement of NETs in the failure of current treatments has also been probed. Numerous studies have demonstrated that NETs might diminish the therapeutic efficacy of targeted therapy and immunotherapy (12–14). In summary, the role of NETs in the area of oncology has attracted enormous attention. However, studies probing the contribution of NETs in ccRCC are few. Even though two previous studies have recently documented the prognostic signature of NETs-related genes for cc-RCC (15, 16), the role of NETs in ccRCC warrants more research. Therefore, conducting a systematic exploration of NETs-related genes in ccRCC is immensely important in comprehending the underlying molecular and signaling pathways of this phenomenon in the development of ccRCC.

This study was carried out with the aim of extensively investigating the attributes of NETs-related genes in ccRCC, in order to gain valuable insights into the function of NET-related genes in the development and prognosis of ccRCC, and to identify potential therapeutic targets for the treatment of ccRCC.

## 2 Materials and methods

### 2.1 Public dataset collection

Data for transcriptome RNA sequencing and clinical information for 598 ccRCC cases (72 normal samples and 526 tumor samples) were sourced from the TCGA (The Cancer Genome Atlas) database (https://xenabrowser.net/datapages/) (17). Cases with clinical data and the overall survival (OS) missing were excluded. Meanwhile, two independent databases: ICGC (International Cancer Genome Consortium) database (https://icgc.org/) containing 91 ccRCC patients; and E-MTAB-1980 database (https://www.ebi.ac.uk/arrayexpress/) (18) comprising 101 ccRCC patients, were retrieved to validate our findings.

### 2.2 Identification of DEGs related to NETs

A group of 69 genes linked with NETs were extracted from prior studies (11, 15, 16, 19, 20) and shown in **Table S1**. The differently expressed genes (DEGs) associated with NETs between tumor and normal tissues were identified using the “DESeq2” package in R. Candidate NETs genes were obtained after setting the fold change (|fold change| ≥ 1) and adj. P < 0.05. The “pheatmap” R package was utilized to generate heatmap of the DEGs. Then, STRING (Search Tool for the Retrieval of Interacting Genes; Version 11.0, https://string-db.org/) was used to acquire a protein −protein interaction (PPI) network.

### 2.3 Construction of a ccRCC prognostic model based on NETs-related genes

Initially, we assessed the prognostic significance of the NETs-associated genes using univariate Cox regression analysis, with a significant filter set at 0.05, employing the “survival” R package. Subsequently, based on the results obtained from the aforementioned analysis, we utilized the “glmnet” R package to develop a prognosis model, utilizing LASSO (least absolute shrinkage and selection operator). The following formula was used to calculate the risk score: risk score = β (1) gene (1) × expression of gene (1) + β (2) gene (2)× expression of gene (2)+…+ β(n) gene(n) × expression of gene(n). Patients were classified into high-risk (>median number) and low-risk (≤median number) groups based on the median value of risk score. The “survival” and “survminer” R package were used to conduct the Kaplan–Meier analysis (21, 22). The R package “survival ROC” was used to examine the predictive accuracy at 1-, 3-, and 5-year intervals through a time-dependent receiver operating characteristic (ROC) curve analysis.

### 2.4 The prognostic value of risk score and built of nomogram

Clinical parameters (age, gender, and tumor stage) of the patients in the TCGA cohort were extracted. Together with the risk score, these clinical variables were analyzed by univariate and multivariate Cox proportional hazards regression. The Schoenfeld residuals were calculated for each variable to assess if each variable independently satisfied the assumptions of the Cox model. By using the “rms” and “survival” R packages, a nomogram was further depicted to predict OS in TCGA patients (1 year, 3 years, and 5 years) based on the previously mentioned NETs signature and clinical prognostic factors. Besides, we have performed the decision curve analysis to compare the prognostic value of models including and not including risk score.

### 2.5 Functional enrichment analyses

The TCGA cohort of ccRCC patients were divided into two subgroups, characterized as per their risk scores. DEGs were separated using FDR<0.01 and |log2FC| ≥ 1.5 filtering criteria between the high- and low-risk group. Subsequently, we executed enrichment analyses (GO and KEGG analysis) with the aid of the “ClusterProfiler” R package to identify potential correlation between the enrichment terms.

### 2.6 Assessment of immune microenvironment

The TCGA patients were divided into two groups according to their risk scores. The assessment of tumor microenvironment for both groups along with stromal score, immune score and ESTIMATE score was conducted using the “ESTIMATE” R package (23). To quantify the immunologic cell abundances in the immune microenvironment, the infiltration levels of 28 immune cell types were evaluated by the enrichment score, which was calculated via the single-sample gene set enrichment analysis (ssGSEA) method from “GSVA” R package (24, 25). Furthermore, we gathered a group of key immune checkpoints and compared their different expressions between two NET risk groups.

### 2.7 Cancer treatment prediction

The GDSC (Genomics of Drug Sensitivity in Cancer) database provides information regarding the sensitivity of cancer cell lines to diverse anticancer drugs (26), which was used to predict drug susceptibility for TCGA-KIRC patients. Drug sensitivity were calculated with the “oncoPredict” R package (27). Then we compared the therapeutic ability of several common targeted therapies in KIRC between two NET risk groups.

### 2.8 Statistical analysis

R software (version 4.3.0) and SPSS software (version 23.0) were employed for all statistical analyses. Box plot analyses were conducted using the Wilcoxon rank-sum test. Kaplan-Meier method was utilized to generate survival curves for high-risk and low-risk groups of patients. The significance of differential groups was assessed using the log-rank test. All hypothetical analyses were two-sided, where a P value < 0.05 was deemed significant.

## 3 Results

### 3.1 Identification of differently expressed NETs-related genes between normal and tumor tissues

**Figure 1:**
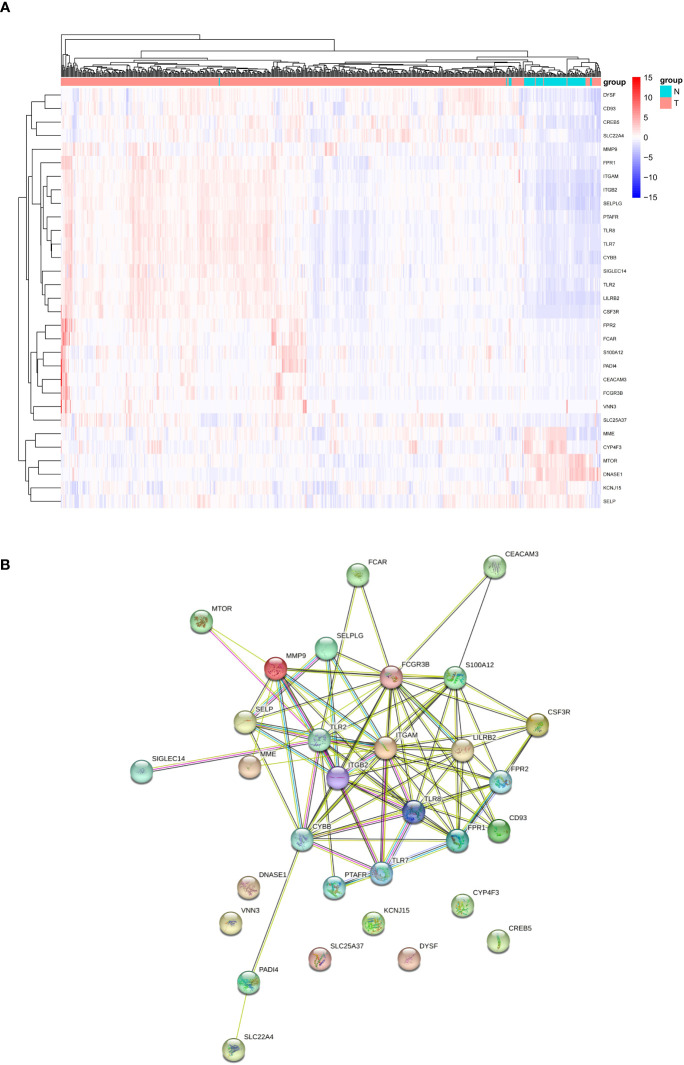
Analyses of DEGs. **(A)** Heatmap of differentially expressed NETs-related genes between tumor and normal samples from TCGA-KIRC cohort. **(B)** Interaction between NETs-related genes by the PPT network. NET, neutrophil extracellular trap; DEGs, differentially expressed genes; PPI: protein −protein interaction.

Expression levels of 69 genes related to NETs were compared between 526 tumors and 72 normal tissues in the database TCGA-KIRC, leading to the identification of 31 DEGs. Six genes (DNASE1, CYP4F3, MME, KCNJ15, MTOR, SELP) were down-regulated and 25 other genes (SLC22A4, SLC25A37, CD93, FCAR, PADI4, FPR2, FCGR3B, S100A12, PTAFR, DYSF, CREB5, FPR1, TLR2, SIGLEC14, CEACAM3, CYBB, ITGAM, SELPLG, TLR7, TLR8, LILRB2, ITGB2, VNN3, CSF3R, MMP9) were up-regulated in tumor tissue samples. RNA expression of these genes was visualized through a heatmap (**Figure 1A**, blue: low level of expression; red: high level of expression). To explore potential interactions among these NETs-related genes, PPI analysis was conducted by applying the STRING platform, and the results were shown in **Figure 1B**.

### 3.2 Construction and validation of the NET risk score for OS prediction

**Figure 2:**
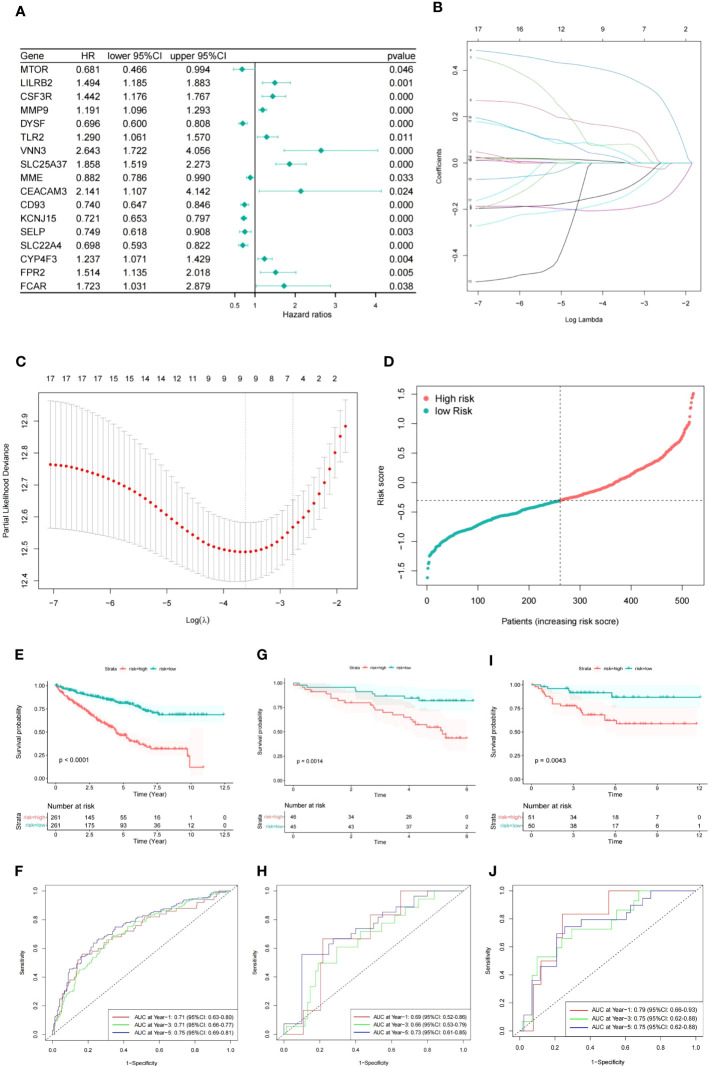
Construction of prognostic signature based on NETs-related genes. **(A)** Forest plot of NETs-related genes with P<0.05. **(B)** Nine genes linked to OS were analyzed by LASSO regression. **(C)** The selection of parameters was cross-validated. **(D)** ccRCC patients distribution according to the risk score. **(E, F)** Kaplan-Meier analysis and corresponding ROC curve for TCGA patients. **(G, H)** Kaplan-Meier analysis and corresponding ROC curve for ICGC patients. **(I, J)** Kaplan-Meier analysis and corresponding ROC curve for E-MTAB-1980 database.

To identify the significant NETs-related genes for TCGA patients, univariate Cox regression analysis was contacted to evaluate their prognostic value. 17 genes were selected after filtered by P<0.05. The forest plot represented the HR of each single gene (**Figure 2A**). LASSO regression analysis was utilized to construct a prognostic model, and nine genes along with their coefficients were ultimately included in the NETs-related gene prognostic signature, based on the optimal λ value. (**Figures 2B, C**). The risk score was calculated as following, NET risk score = 0.003 × MMP9 + 0.08 × VNN3 + 0.386 × SLC25A37 - 0.154 × CD93 -0.201 × KCNJ15 - 0.121 × SLC22A4 + 0.152 × LILRB2 + 0.049 × TLR2 + 0.020 × FPR2. Then, the 519 TCGA patients were classified into high- and low- risk groups based on the median value of risk score (**Figure 2D**). The Kaplan-Meier curves demonstrated that patients in the high-risk group had a significantly shorter OS compared to those in the low-risk group (**Figure 2E**, P<0.0001), and the AUCs for 1-, 3- and 5-year OS rates were found to be 0.71, 0.71 and 0.75, respectively (**Figure 2F**).

Subsequently, we used ICGC and E-MTAB-1980 cohorts as two independent validation datasets and observed a similar outcome in the high risk group of ccRCC patients (**Figures 2G–J**).

To assess the independence of NET risk score, the univariate and multivariate Cox regression analyses for the NETs-related signature and other clinical pathological factors associated with OS were performed. Results were presented in **Table 1**, revealing that the risk score was deemed an independent risk factor for the prognosis of TCGA patients with ccRCC (HR: 2.53, 95% CI: 1.77–3.61, P <0.001). Besides, results from the Schoenfeld residual revealed that each variable in the multiple Cox model independently satisfied the assumptions of the Cox model (**Figure S1**).

**Table 1:** Univariate and multivariate Cox regression analyses of the signature risk score and other clinical variables for OS in the TCGA-KIRC cohort.

Variables	No. (%)	Univariate		Multivariate	
		HR(95%CI)	P value	HR(95%CI)	P value
Age	61 (26.6-88.7)	1.03 (1.02-1.04)	<0.001	1.03(1.01-1.04)	<0.001
Gender		0.94 (0.68-1.28)	0.68		
Female	181 (35%)				
Male	337 (65%)				
Tumor stage		1.90 (1.67-2.18)	<0.001	1.75(1.52-2.01)	<0.001
Stage I	259 (50%)				
Stage II	56 (11%)				
Stage III	121 (23%)				
Stage IV	82 (16%)				
Risk score		3.56 (2.52-5.02)	<0.001	2.53(1.77-3.61)	<0.001
Low	259 (50%)				
High	259 (50%)				

By comparing the AUC among different models, we can determine whether the NET risk signature in this study improves the performance in terms of OS prediction. ROC analysis showed that the model comprised of NET risk signature and clinical variables (age and stage) have the higher AUC value at 1-, 3- and 5-year than the other model only including clinical variables, confirming the valuable contribution of NET risk signature (**Figure S2**).

In addition, Kaplan–Meier survival curves for nine NETs genes and correlation between their expression and clinical characteristics were presented in **Figures S3**, **S4**, respectively, to shed light on the role of prognostic signature genes in the development of ccRCC. In brief, except KCNJ15 was down regulated in tumor, the other eight genes were all up regulated. KCNJ15 together with CD93 and SLC22A4 were decreasingly expressed in the higher grade of tumor. Patients with low expression of KCNJ15, CD93 and SLC22A4 had shorter OS. All together, KCNJ15 may act as a tumor suppressor in ccRCC progression.

### 3.3 Nomogram construction

**Figure 3:**
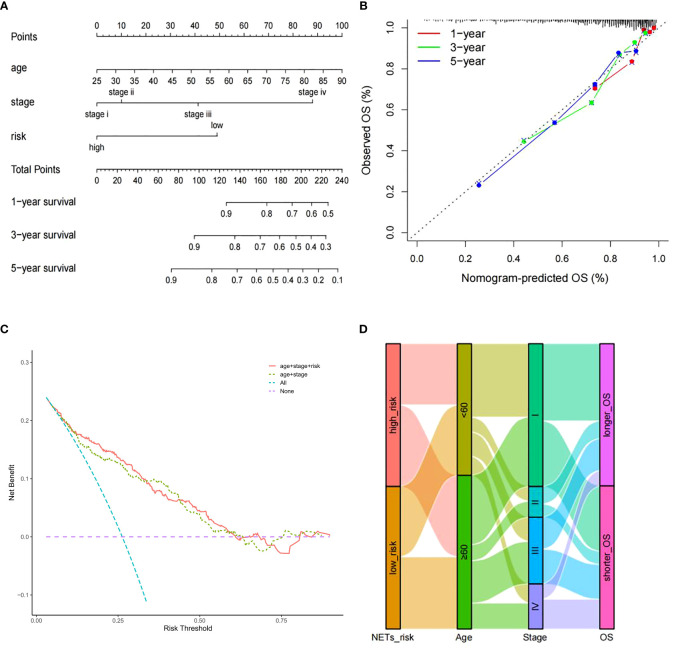
Nomogram construction. **(A)** Nomogram for predicting 1-, 3-, or 5-year OS rates in patients with ccRCC. **(B)** Calibration plots for predicting 1-, 3-, or 5-year OS rates.**(C)** Decision curve analysis comparing the prognostic value of different models. **(D)** Sankey Diagram showing the association between signature risk scores and clinical pathological characteristics.

To enhance the predictive capacity of the signature for ccRCC patients, a nomogram developed by incorporating available clinical pathological parameters and risk score. Through the multivariate Cox regression analysis, age, stage, and risk score were considered as independent factors and were included in the nomogram (**Figure 3A**). Moreover, calibration curves were plotted for 1-, 3-, and 5-year survival rates to assess the accuracy of the nomogram (**Figure 3B**). The conspicuous agreement emerged for the OS predicted by the nomogram and the reliable values across the following period. The stability and accuracy of the nomogram including our NETs-related genes signature as well as age and tumor stage can predict the outcome of individual patients. In addition, the decision curve model showed that the prognostic model with risk score had a good performance (**Figure 3C**). The association between signature risk scores and clinical pathological characteristics was presented in the form of a Sankey diagram (**Figure 3D**). A higher signature risk score was significantly correlated with higher tumor stage (p < 0.001).

### 3.4 Functional enrichment analysis

**Figure 4:**
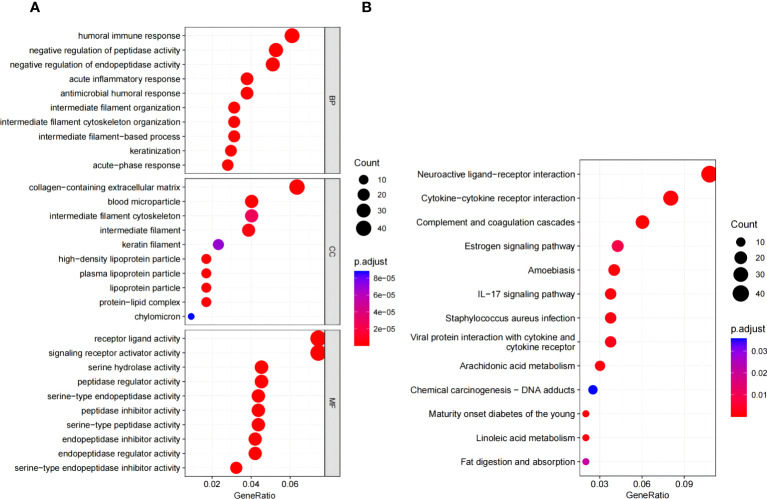
Functional enrichment analysis. **(A)** Go enrichment analysis of DEGs. **(B)** KEGG pathway enrichment analysis of DEGs. GO, gene ontology; KEGG, Kyoto encyclopedia of genes and genomes.

GO and KEGG analyses were conducted to assess the biological involvement of the DEGs. As presented in **Figure 4A**, the top GO terms comprised acute inflammatory response, lipid catabolic process, collagen-containing extracellular matrix, etc. Furthermore, KEGG analysis indicated that the DEGs were primarily involved in pathways such as complement and coagulation cascades, staphylococcus aureus infection and estrogen signaling pathway, as shown in **Figure 4B**.

### 3.5 Disparate immune microenvironment and potential immunotherapies and targeted therapies response between low and high NET risk groups

**Figure 5:**
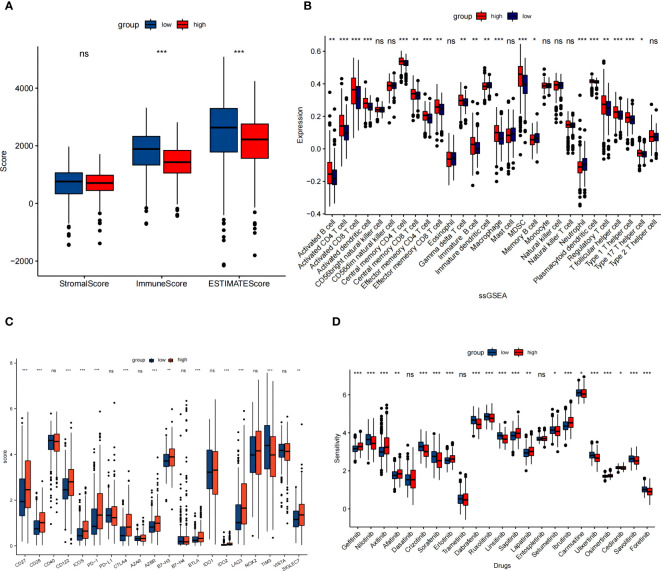
NET risk scores in relation to immune microenvironment in the TCGA cohort. **(A)** Changes in ESTIMATE among KIRC patients with high and low NET scores. **(B)** Box plot showed the different expression of tumor infiltrating cells between different groups based on ssGSEA algorithm. **(C)** The expression levels of immune checkpoints between high and low risk groups. **(D)** Box plot of estimated drug sensitivities for several GDSC target therapeutics in the high and low NET scores groups. ns, no significance. *p < 0.05, **p < 0.01, ***p < 0.001.

The association between NET risk classification and the immune status in patients with KIRC was assessed. We found that KIRC patients in low risk group had notably elevated ESTIMATE scores and immune scores when compared to those in high risk group (**Figure 5A**). These results suggest an inverse relationship between NET scores and immune status in KIRC patients. Then, we compared the immune cell infiltration in the high versus low NET risk groups based on”ssGSEA” algorithm. The findings revealed that the immunosuppressive cells, such as myeloid-derived suppressor cells (MDSC) and regulatory T cells (Tregs), were significantly higher in the NET high risk group (**Figure 5B**). This phenomenon may imply the immunosuppressive microenvironment in the NET high risk group. In addition, the role played by the 9 NETs genes in immune cell infiltration were displayed separately (**Figure S5**).

We figured out the disparate expression of immune checkpoints between low and high NET risk groups. Except TIM-3, the expression of majority selected immunosuppresive checkpoints PD-1, CTLA4, LAG3, A2BR, and B7-H3 were all significantly upregulated in the high risk group (**Figure 5C**). Moreover, a significant association between NETs risk group and the efficacy of targeted therapies such as gefitinib, axitinib, afatinib, erlotinib, saptinib, and ibrutinib was detected. Specifically, patients with lower NETs-scores had a better response to these targeted therapies (**Figure 5D**).

## 4 Discussion

Despite breakthrough development in comprehensive therapy of ccRCC in recent years, treatment outcome for ccRCC patients varies individually. Several clinical factors and gene signature have been identified as potential predictors for ccRCC. Even though, the predictive ability is insufficient. NETs is a newly detected type of programme cell death specifically induced by neutrophil. This process has been initially shown to play a key role in the development of immune-related disease such as chronic inflammation (28). Recently NETs has been recognized to influence tumor growth, metastasis as well as treatment outcome (13, 29–31). MMP-9 derived from neutrophils has been shown to be associated with VEGF activation and angiogenesis, which are generally hallmark of ccRCC (32). Cools-Lartigue J et al. have found that NETs can promote hepatic metastasis by aiding the survival and growth of lung carcinoma cells (30). Some studies have freshly explored the important involvement of certain NETs-related genes in cancers (15, 16, 33, 34). However, the precise impact of NETs on the development and prognosis of ccRCC have yet to be fully understood. Accordingly, we conduct this study to comprehensively explore the role of NETs-related genes in ccRCC patients using the data from public datasets.

In the present study, we analyzed expression of 69 NETs-related genes in normal and ccRCC tissues from TCGA dataset, and found that 31 of them were differently expressed. Next, the prognosis value of these genes were examined, and 17 were significantly associated with overall survival. After LASSO Cox regression analysis, nine NETs-related genes (MMP9, VNN3, SLC25A37, CD93, KCNJ15, SLC22A4, LILRB2, TLR2 and FPR2) were enrolled to construct the NETs prognosis signature. All the ccRCC patients from TCGA were correspondingly divided into low and high risk group. Our findings suggest that patients in high risk group had a worse prognosis with shorter overall survival. The AUCs for 1-, 3- and 5-year OS rates were 0.71, 0.71 and 0.75, respectively, implying the good performance of the NETs prognosis signature. Similar results was confirmed in two validation cohorts.

Of these nine core genes set, MMP9 has been investigated extensively in ccRCC. As it promotes angiogenesis, facilitates tumor cell invasion and migration (32), the elevated MMP9 levels have been confirmed to correlate with tumor grade, stage, and poor prognosis of ccRCC (35, 36). SLC22A4 and SLC25A37 are members of the solute carrier (SLC) gene family encoding membrane transporters that play an important role in anticancer drug resistance (37). Till now, there is no consensus on the function of SLC22A4 in tumor. Buelow et al. have reported that increased basal SLC22A4 methylation was associated with decreased cytarabine in acute myeloid leukemia, resulting in cytarabine resistance (38). On the other hand, Okabe et al. have found that over-expression of SLC22A4 can increase cellular uptake and heighten sensitivity to mitoxantrone and doxorubicin (39). SLC25A37 has not yet been fully investigated. In our research, its over-expression was associated with poor prognosis, which was consistent with previous study (40). In the established NETs signature, KCNJ15 was the only downregulated gene in ccRCC tissue. Moreover, KCNJ15 may be a tumor suppressor since its over-expression significantly inhibited RCC cell proliferation, migration, and colony formation through regulating epithelial mesenchymal transition process (41). Beyond aforementioned genes, VNN3, CD93, LILRB2, TLR2 and FPR2 have been included as prognosis biomarker in various type of tumor including ccRCC (42–46).

Additionally, we compared the immune infiltration between the low and high risk group according to NETs prognosis signature. The high risk group with poorer survival was linked with elevated level of specific immune cell types, for instance, Tregs and MDSC. This phenomenon was consistent with the aforementioned study by Senbabaoglu et al, in which the accumulation of regulatory T cells correlated with worse survival (20). In addition to Tregs, MDSC was generally considered to promote tumor angiogenesis and metastasis of RCC, and therefore, the accumulation of MDSC was negatively related to survival (47). These results suggest that NETs formation may influence the immune environment.

Finally, we observed the significant association between NETs risk group and the efficacy of targeted therapies and immunotherapies. Regarding to checkpoint inhibitors, patients with low NETs risk score and higher expression levels of PD1, CTLA-4 and LAG3 are more likely to have benefit from immunotherapies. We assessed 22 kinase inhibitors using the GDSC2 dataset and found that gefitinib, axitinib, afatinib, erlotinib, saptinib, and ibrutinib treatments may be beneficial for patients with advanced ccRCC who have a low NET-score.

The findings of this study have potential clinical implications, as they suggest that a NET signature could be used as a prognostic biomarker in ccRCC. This could inform treatment decisions and help to identify patients who are at a higher risk of disease progression or mortality. Additionally, the association between the NETs signature and specific immune cell types suggests that targeting NETs formation could be a potential therapeutic strategy in ccRCC. However, it is important to note that this study was based on bioinformatic analysis and will need to be validated in further clinical studies before the results can be translated into clinical practice.

## 5 Conclusions

This study provides valuable insights into the potential role of NETs in ccRCC. The findings suggest that a NETs signature could have clinical relevance as a prognostic biomarker and potential therapeutic target. However, further studies will be needed to validate these findings and determine the broader implications of NETs formation in cancer.

## Data availability statement

The datasets presented in this study can be found in online repositories. The names of the repository/repositories and accession number(s) can be found below: The accession number for TCGA is phs000178.v11.p8. And all the data were downloaded from https://xenabrowser.net. The accession number for ICGC is RECA-EU. Relevant data were obtained from https://dcc.icgc.org/.

## Ethics statement

Ethical review and approval was not required for the study on human participants in accordance with the local legislation and institutional requirements. Written informed consent for participation was not required for this study in accordance with the national legislation and the institutional requirements.

## Author contributions

RL and XL designed this study and conceptualized the methodology; PW and XJ analyzed the data; XL performed the validation; RL wrote the draft manuscript; PW, XJ, and XL revised the manuscript. All authors contributed to the article and approved the submitted version.
