# Supplementary material for: Prognostic value of neutrophil extracellular trap signature in clear cell renal cell carcinoma
Source: Front Oncol. 2023 Jul 13;13:1205713. doi: 10.3389/fonc.2023.1205713 (PMC10374836; doi:10.3389/fonc.2023.1205713)
Supplement: Supplementary file 1 [file DataSheet_1.pdf]

FIGURE S1 The Schoenfeld residuals curve for each variable in the Cox model.

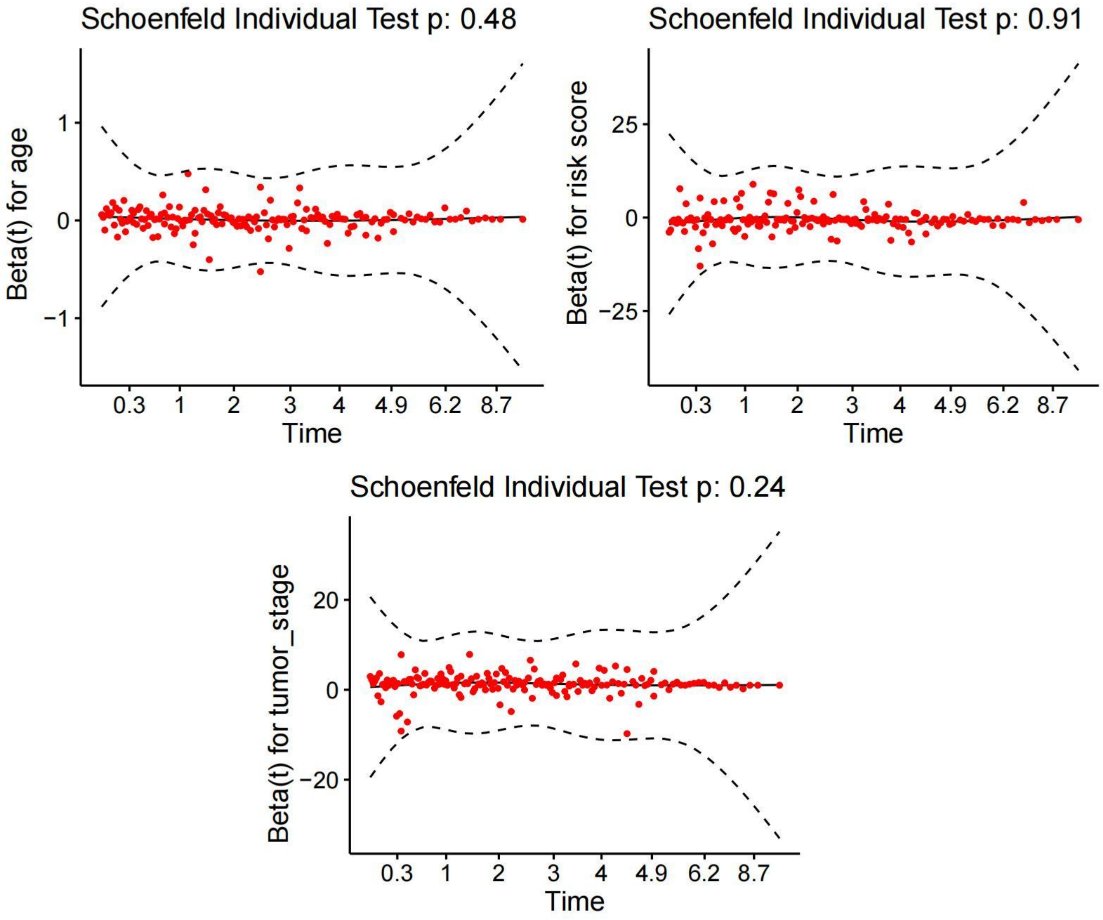

FIGURE S2 ROC analysis comparing different models. (A)AUC prediction at 1-year; (B) AUC prediction at 3-year; (C) AUC prediction at 5-year.

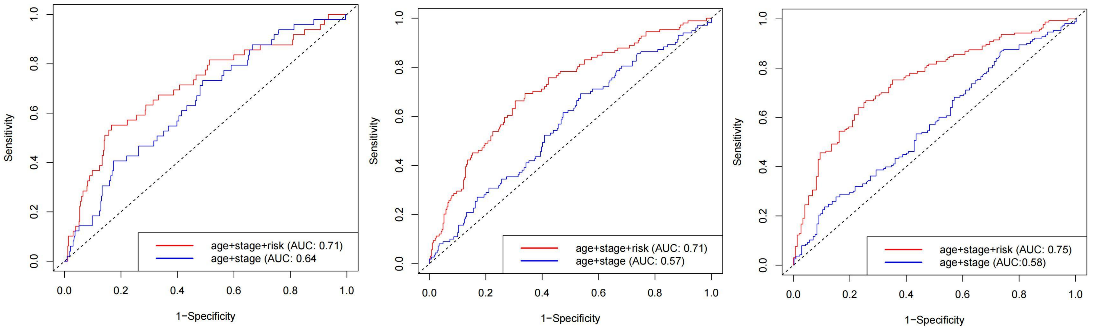


(A) (B) (C)

FIGURE S3 Kaplan-Meier curves for nine NETs genes in TCGA patients

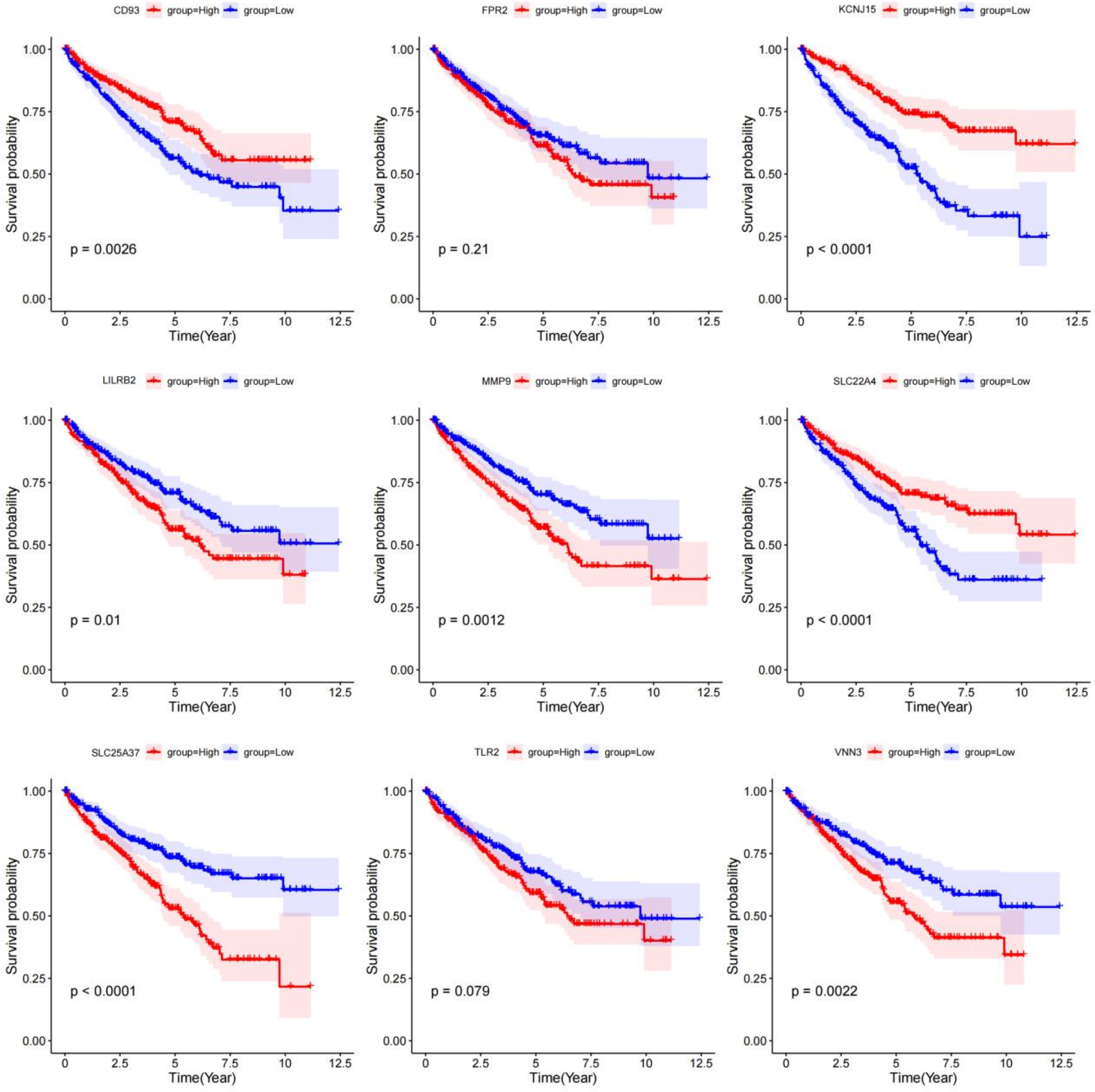

FIGURE S4 Prognostic signature gene analysis. (A) Different expression between tumor and normal. Correlation between the pT stage (B), pN stage (C), pM stage (D), tumor grade (E). *p < 0.05; **p < 0.01; ***p < 0.001

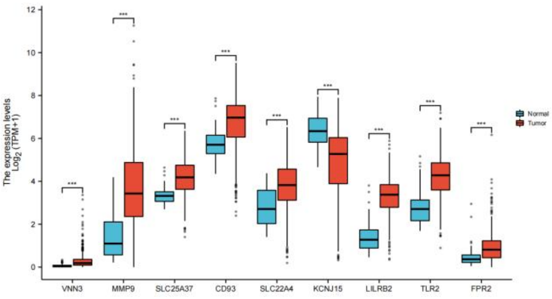


(A)

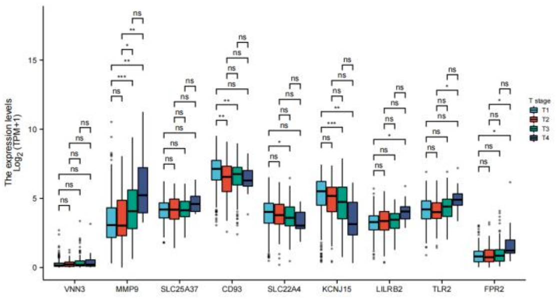


(B)

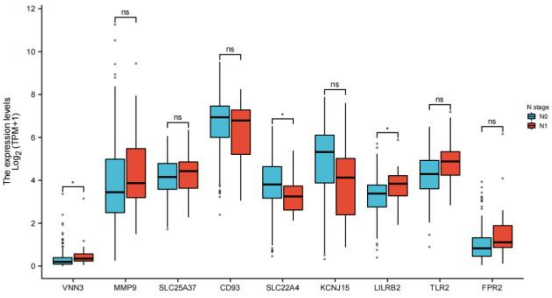


(C)

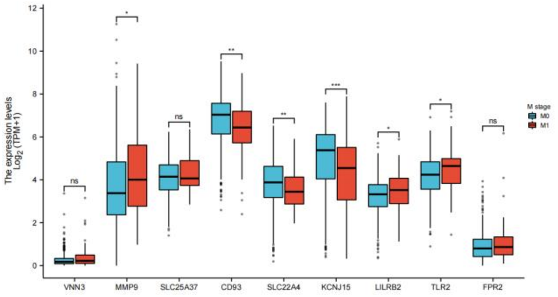


(D)

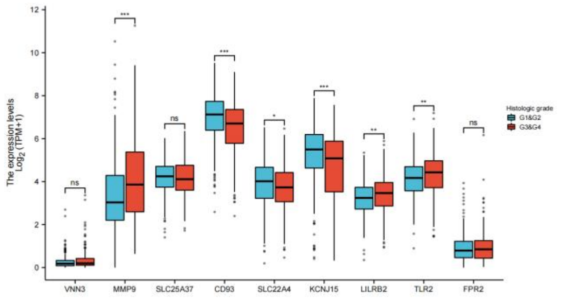


(E)

FIGURE S5 Correlation of nine NETs genes with immune infiltration levels in ccRCC.

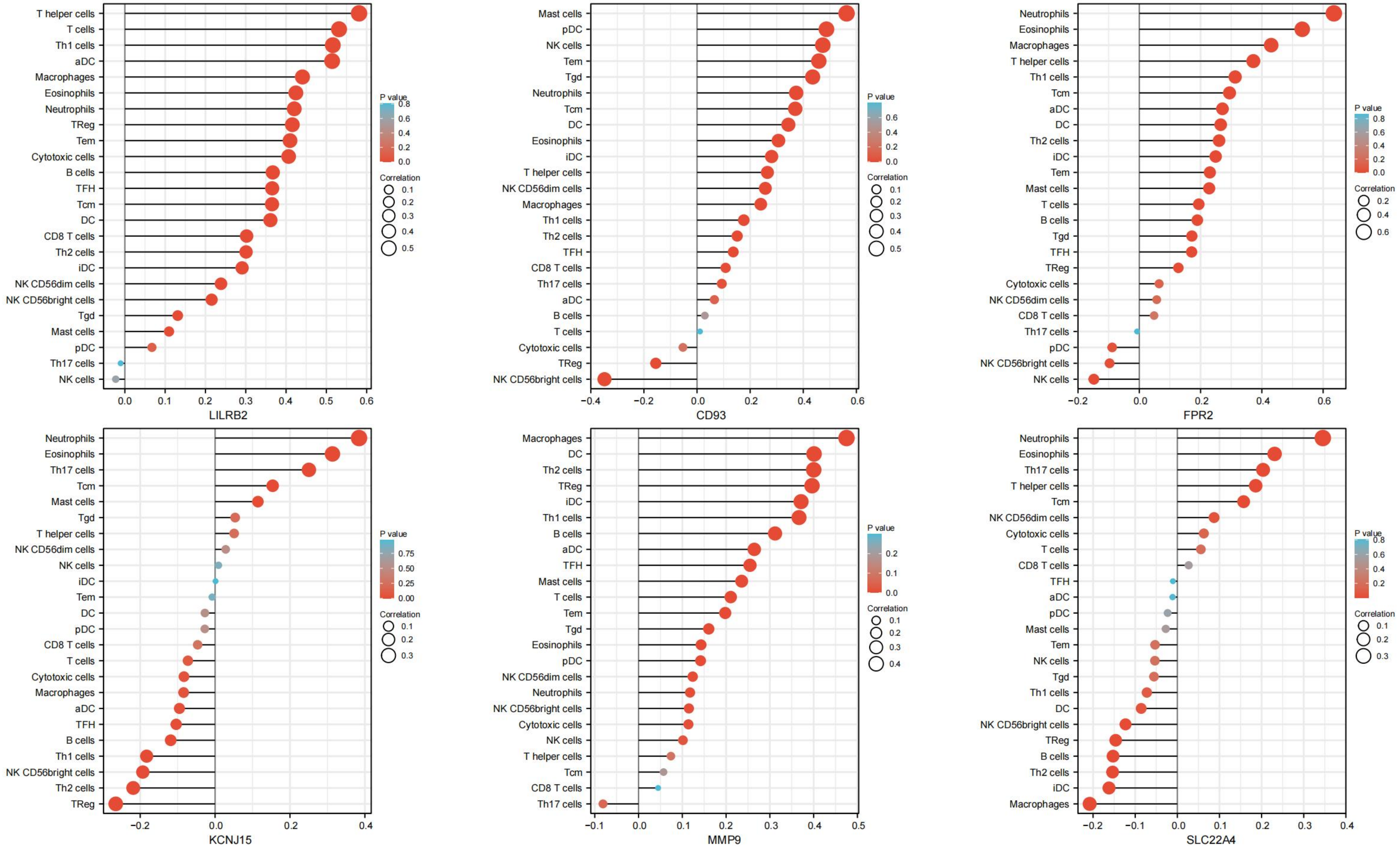

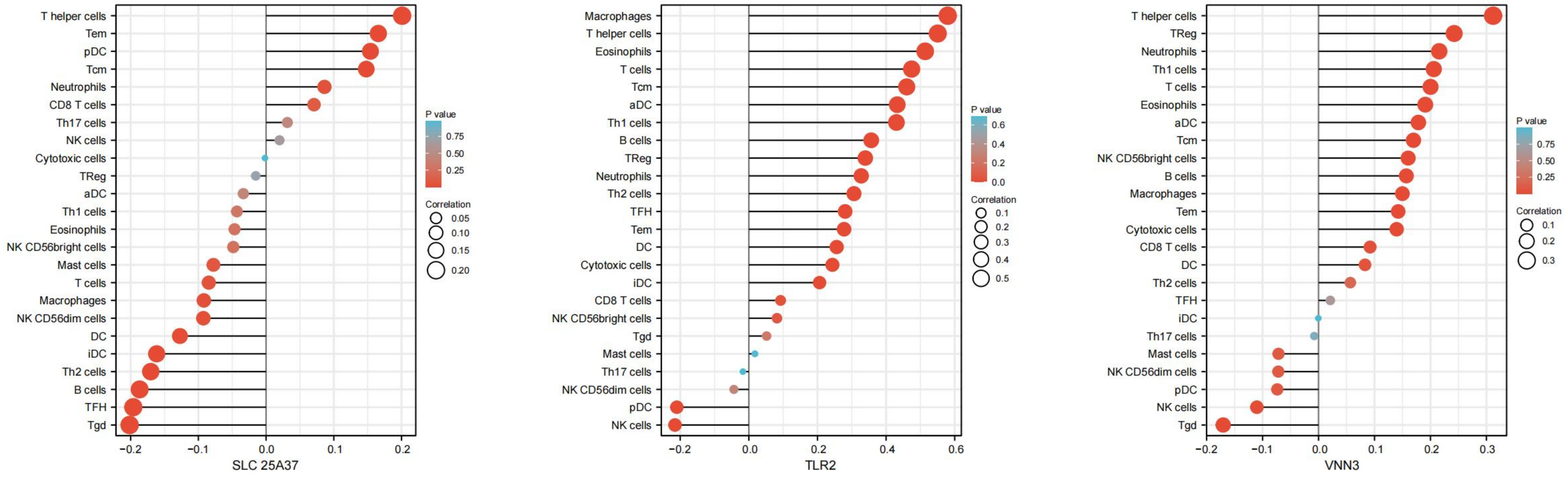
T helper cells
Tem
pDC
Tcm
Neutrophils
CD8 T cells
Th17 cells
NK cells
Cytotoxic cells
TReg
aDC
Th1 cells
Eosinophils
NK CD56bright cells
Mast cells
T cells
Macrophages
NK CD56dim cells
DC
iDC
Th2 cells
B cells
TFH
Tgd
−0.2 −0.1 0.0 0.1 0.2
SLC 25A37
P value
0.75
0.50
0.25
Correlation
0.05
0.10
0.15
0.20
Macrophages
T helper cells
Eosinophils
T cells
Tcm
aDC
Th1 cells
B cells
TReg
Neutrophils
Th2 cells
TFH
Tem
DC
Cytotoxic cells
iDC
CD8 T cells
NK CD56bright cells
Tgd
Mast cells
Th17 cells
NK CD56dim cells
pDC
NK cells
−0.2 0.0 0.2 0.4 0.6
TLR2
P value
0.6
0.4
0.2
0.0
Correlation
0.1
0.2
0.3
0.4
0.5
T helper cells
TReg
Neutrophils
Th1 cells
T cells
Eosinophils
aDC
Tcm
NK CD56bright cells
B cells
Macrophages
Tem
Cytotoxic cells
CD8 T cells
DC
Th2 cells
TFH
iDC
Th17 cells
Mast cells
NK CD56dim cells
pDC
NK cells
Tgd
−0.2 −0.1 0.0 0.1 0.2 0.3
VNN3
P value
0.75
0.50
0.25
Correlation
0.1
0.2
0.3
